# Cardiac Troponin T Release after Football 7 in Healthy Children and Adults

**DOI:** 10.3390/ijerph17030956

**Published:** 2020-02-04

**Authors:** Rafel Cirer-Sastre, Alejandro Legaz-Arrese, Francisco Corbi, Isaac López-Laval, Juan José Puente-Lanzarote, Vicenç Hernández-González, Joaquin Reverter-Masia

**Affiliations:** 1 National Institute of Physical Education of Catalonia (INEFC), University of Lleida (UdL), Partida la Caparrella s/n, E-25192 Lleida, Spain; fcorbi@inefc.es; 2 Research Group Human Movement (RGHM), Universitat de Lleida (UdL), Plaça de Víctor Siurana, 25003 Lleida, Spain; vicens_h_g@didesp.udl.cat (V.H.-G.); reverter@didesp.udl.cat (J.R.-M.); 3 Section of Physical Education and Sports, University of Zaragoza, Calle de Pedro Cerbuna, 50009 Zaragoza, Spain; alegaz@unizar.es (A.L.-A.); isaac@unizar.es (I.L.-L.); 4 Lozano Blesa University Hospital, Avda. San Juan Bosco, 50009 Zaragoza, Spain; jjpuentel@gmail.com; 5 Section of Physical Education, Universitat de Lleida (UdL), Plaça de Víctor Siurana, 25003 Lleida, Spain

**Keywords:** cardiac biomarkers, exercise physiology, maturation

## Abstract

The objective of this study was to compare the release of cardiac troponin T (cTnT) after a football 7 match between two cohorts of children and adult players. Thirty-six male football players (children = 24, adult = 12) played a football 7 match, and cTnT was measured before, and 3 h after exercise. Concentrations of cTnT were compared between groups and time, and correlated with participants’ characteristics, as well as internal and external exercise load. Cardiac troponin T was elevated in all participants (*p* < 0.001), and exceeded the upper reference limit for myocardial infarction in 25 (~70%) of them. Baseline concentrations were higher in adults (*p* < 0.001), but the elevation of cTnT was comparable between the groups (*p* = 0.37). Age (*p* < 0.001), body mass (*p* = 0.001) and height (*p* < 0.001), and training experience (*p* = 0.001) were associated to baseline cTnT values, while distance (*p* < 0.001), mean speed (*p* < 0.001), and peak (*p* = 0.013) and mean (*p* = 0.016) heart rate were associated to the elevation of cTnT. The present study suggests that a football 7 match evoked elevations of cTnT during the subsequent hours in healthy players regardless of their age. However, adults might present higher resting values of cTnT than children. In addition, results suggest that the exercise-induced elevations of cTnT might be mediated by exercise load but not participant characteristics.

## 1. Introduction

The release of cardiac troponins (cTn), typically observed in patients with acute and chronic myocardial injury (MI), is also common in healthy athletes of all ages and training statuses after exercise [1,2]. This subject is potentially important for the triage of athletes who develop chest pain that mimics cardiac injury after exercise and who might have serum cardiac troponins drawn in the emergency room. The relationship between age and the magnitude of exercise-induced elevations of cTn, however, is still controversial, especially when comparing adolescent with adult athletes [3,4,5]. Whilst some previous studies reported higher peak cTn values in adolescents when compared with the adults [5], others found no age differences [3,4]. Despite this, studies focusing on this phenomenon in younger populations are still scarce [6]. It has been hypothesized that, the higher elevations of cTn in young athletes might be related to maturation, since the immature myocardium might be more vulnerable to injury in clinical situations [3,5]. Since knowledge about the expected blood concentration of cTn in healthy active children after exercise is still limited, this makes it difficult to differentiate physiological and pathological elevations of cardiac troponin T (cTnT) in young patients involved in exercise in the hours previous to a blood analysis [7]. 

This release has been described in a variety of sports, and was related to exercise load [8]. However, although football is the most practiced sport in the world [9], only two studies, to the best of our knowledge, provided data about cTn elevations after its practice in young players [10,11]. Hosseini et al. investigated the elevations of cTn after a football 11 match in a cohort of 22 adolescents [11], while Cirer-Sastre et al., described the associations between exercise load in a small-sided 5-by-5 game and the subsequent elevations of cTn in a cohort of 20 adolescents [10]. Despite this, football matches in early age categories are normally played in smaller teams and pitches [12], usually under regional adaptations of the international rules of football 7, where football matches are played in teams of seven players [13]. To date, no evidence has been reported about cTnT elevations after a football 7 match in young players.

For these reasons, the purpose of this study was to compare the release of cTnT after a football 7 match in two cohorts of children and adult players. Our main hypothesis was that cTnT would be elevated over time and this elevation might vary between age groups and be associated with participant characteristics and exercise load.

## 2. Materials and Methods

### 2.1. Participants

Twenty-four children and 12 adults volunteered to participate in this study after an open invitation at the beginning of the season. All players were male, trained 3 days per week in the same local football club (Peñiscola, Spain), were non-smokers and were not under medical, pharmacologic or dietary treatment. The participants were informed of the purpose, procedures and risks of this study, and gave their prior personal (and parental for those under the age of 18) written informed consent to participate in this study. The inclusion criteria were male, at least 3 years of experience in competitive football, no previous history of cardiovascular disease and favorable readiness according to the revised Physical Activity Readiness Questionnaire (PAR-Q) [14]. No exclusions were made. The procedures of this study were approved by the Ethical Committee of Clinical Research of the Sports Administration of Catalonia (02/2018/CEICGC) and met the principles of the latest revision of the Declaration of Helsinki [15].

### 2.2. Procedures

The participants were required to avoid exercise during the 24 h before the study. On arrival, players underwent a resting 12-lead electrocardiogram (ECG; Click ECG BT 12 channel, Milano, Italy). Then, body mass and height were measured with a medical scale and stadiometer (SECA 711, Hamburg, Germany; and Año-Sayol, Barcelona, Spain; respectively). Subsequently, participants were equipped with a heart rate (HR) chest band (Garmin, Ltd., Olathe, KS, USA) synced with a global positioning system (GPS) tracker (RealTrack Systems, Almería, Spain). This set up has been previously validated for both heart rate [16] and geospatial tracking [17]. Participants performed a standardized “11+ Kids” warm-up [18] and then played a 7 × 7 match (2 children’s matches, 1 adults’ match) following the rules of the International Federation of Football 7 [13]. The match time was divided in two parts separated by half time of 10 min, and each part consisted of two quarters of 15 min with a 2 min break between quarters. All participants played the complete match, no player replacements were made and goalkeepers were excluded from data analysis.

Blood samples were taken from an antecubital vein before exercise (pre) and at 3 h after exercise (post). The election of these sampling times was based on previous research reporting an approximate time-to-peak for cTnT of between 2 and 5 h after exercise [6]. Blood samples were quickly centrifuged and stored at −80 °C for further analysis. Cardiac Troponin T was determined using a Troponin T hs STAT immunoassay in a Cobas E 601 analyzer (Roche Diagnostics, Penzberg, Germany). The detection range of this assay is 3–10,000 ng/L, and the intra-assay coefficient of variation at a mean cTnT of 13.5 ng/L is <10%. The upper reference limit (URL) for cTnT, defined as the 99th percentile of healthy participants, is 13.5 ng/L [19].

The response variable in this study was the participants’ blood concentration of cTnT, and the main predictors were time (pre; post) and group (children; adults). A variable called delta (∆cTnT) was calculated by subtracting individual pre to post cTnT values. The secondary predictors were participant characteristic data and exercise load. Participant characteristic predictors were age (years), body height (cm) and mass (kg), previous training (years), training frequency (days/week) and training volume (hours/week). The exercise load covariables, obtained during the match, were distance (m), peak speed (SPpeak, km/h), mean speed (SPmean, km/h), peak HR (HRpeak, bpm), peak relative HR (rHRpeak, % HRmax), mean HR (HRmean, bpm), and mean relative HR (rHRmean, % HRmax). The maximum HR (HRmax) for relative calculations was age-predicted using the formula 208.609 − 0.716 × age [20]. 

### 2.3. Statistical Analysis

All statistical analyses were performed using R v3.6.1 (R Foundation for Statistical Computing, Vienna, Austria). A Shapiro–Wilk test and data visualization confirmed that data distribution for cTnT was right-skewed and non-transformable. Homogeneity of variances was assessed using a Flinger–Killeen test (χ^2^). The data were described using mean ± standard deviation and median (range) as appropriate. Between-group comparisons for participant characteristics and performance data were made using an independent-samples *t*-test (*t*). Between-group differences for cTnT were tested using an independent 2-group Wilcoxon rank sum test with continuity correction (*W*). Within-subject differences over time were assessed using dependent 2-group Wilcoxon signed rank tests with continuity correction (*V*). The rates of positive detection for participants with cTnT above the URL were compared using a 2-sample test for equality of proportions with continuity correction (χ^2^). Associations between cTnT and participant characteristics or exercise load variables were assessed using Spearman’s correlation (*r*_s_). Statistical significance for all hypothesis tests was assumed when *p* < 0.05. 

## 3. Results

### 3.1. Participants

The participant characteristics and exercise load are summarized in Table 1. Although there were group differences for age (*p* < 0.001), body mass (*p* < 0.001), height (*p* < 0.001), and years of training experience (*p* = 0.007), the participants underwent comparable training frequency (*p* = 0.73) and volume (*p* = 0.55) at the time of the study. During the match, both groups covered similar distances (*p* = 0.17); however, adults performed at a higher average and peak speed than children (*p* < 0.001 and *p* = 0.04, respectively). By contrast, the absolute peak heart rate (HRpeak) was higher in the children (*p* < 0.001). Besides that, the relative peak heart rate (rHRpeak), absolute average heart rate (HRaver) and relative average heart rate (rHRaver) were comparable between groups (*p* = 0.14, *p* = 0.5 and *p* = 0.084, respectively).

### 3.2. Cardiac Troponins

**Figure 1:**
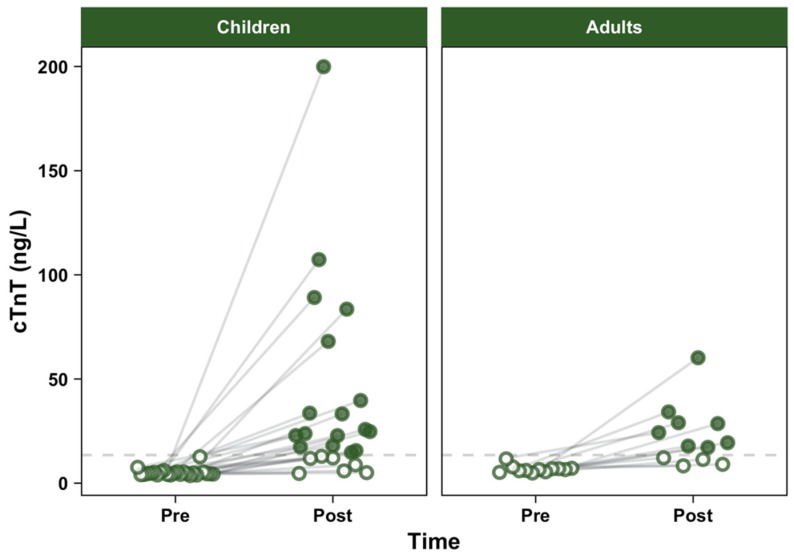
Individual values of cardiac troponin T (cTnT) before and after the match, by group.

Children had lower resting values of cTnT than adults (*p* < 0.001). Both groups had elevated cTnT 3 h after the match (children: *p* < 0.001, adults: *p* = 0.001). Nevertheless, both the elevation and the absolute post-match concentration were comparable between cohorts (*p* = 0.37 and *p* = 0.65, respectively) (Table 2). Although data visualization suggested that children’s cTnT was more variable than adults (Figure 1), both variances were statistically comparable (χ^2^ = 2.53, *p* = 0.11). None of the participants exceeded the URL at baseline. However, 17 (70.83%) children and 8 (66.67%) adults exceeded the cut-off value for MI of 13.5 ng/L at 3 h post exercise. There were no differences between the rates of positive detection (*p* = 0.99).

Correlations between basal values of cTnT, delta cTnT changes, participant characteristics and performance are detailed in Table 3. Baseline concentrations correlated positively with age (*p* < 0.001), body mass (*p* = 0.001), body height (*p* < 0.001), and years of previous experience (*p* = 0.001). Post-exercise elevations were associated with distance (*p* < 0.001), mean speed (*p* < 0.001), peak (*p* = 0.013) and mean (*p* = 0.016) heart rate.

## 4. Discussion

This is the first study comparing the release of cTnT after a football 7 match between two cohorts of children and adult players. Our main findings were that (i) basal cTnT might be higher in the adults, (ii) exercise induces elevations of different magnitude in all players, regardless of their age, (iii) both the absolute post values and the relative increase (∆cTnT) are comparable between children and adults, (iv) participant characteristics were associated with baseline cTnT, but not with its elevation, and (v) exercise load was positively associated with the subsequent cTnT elevation.

Our results are confirmatory of the acute elevation of cTnT in the hours after exercise [2]. As previously noticed in other studies [3,4], participants had normal ECG and reported no history of personal nor familiar cardiovascular disease. However, the overall rate of positive detection was substantial (25/36, 69.44%), and similar to that in a recent meta-analysis including studies with participants under the age of 18 (166/219, 75.8%, χ^2^ = 0.37, *p* = 0.54) [6]. These findings reinforce the hypothesis that transient elevations of cTn after practicing sport might be a physiological acute response to exercise rather than a pathological sign of cardiovascular disease. Therewith, this implies that exercise might confound clinical cTn assessments of patients attending emergency departments, and should be considered during the anamnesis [21].

Another finding in this study was that adult players presented higher basal values of cTnT than children, despite having normal ECG and being apparently healthy. Such a difference coincides with previous studies reporting higher population values in the older cohort [22]. This difference might be explained by age in itself, since it is one of the main risk factors for cardiovascular disease (CVD) [23]. Moreover, older individuals are also more likely to have concealed CVD that might not be detectable by only assessing a resting ECG, as we did in this study [22,24]. Nevertheless, this difference at baseline was not maintained through time, but it disappeared when comparing post cTnT concentrations as well as ∆cTnT.

Yet another relevant finding in this study is the negative result when testing for between-group differences in ∆cTnT. Although some previous studies found higher cTn elevations in adolescents when compared with adults [5], ours is not the first study that failed to find this association [3,4]. These previous studies, however, used adolescent players while ours included children. A higher and more variable release of the biomarker during puberty might explain why some differences were found in adolescent–adult comparisons but not in children–adult studies. This hypothesis goes in line with our recent study in which we found that in a cohort of 20 participants of 11.9 ± 2 years, the elevation of cTnT after exercise was positively associated with the maturational stage of participants obtained using a Tanner scale [10]. In this regard, previous authors have already highlighted the need for age- and sex-specific population values for cTnT concentrations in pediatrics [25]. The present results, therefore, contribute to a better description of how normal and exercise-induced values might vary through age, and evidence the need to stratify population reference values by age. 

In this study, no associations between participant characteristics and the cTn response to exercise were found (Table 3). Several preceding studies reported that ∆cTnT might be partially explained by age [5,10,26], anthropometrics [11] and previous training experience [26,27], while others obtained non-conclusive associations [11,28,29,30]. These discrepancies might be explained by a variety of methodological differences between studies, such as the sample age range, athletic status, exercise mode or the intensity and duration in the interventions. In this regard, although our results support the assertion that the exercise-induced elevation of cTn might be independent of age, height, weight or experience, larger studies including wider age ranges are still needed to clarify this association. Weekly training frequency and volume at the time of the study, on the other hand, were not associated with ∆cTnT either, coinciding with previous reports [31].

Exercise load has previously been correlated with ∆cTnT [8,10]. Similarly, players in this study who covered larger distances, ran at a higher speed, or performed at higher HR were those who presented a higher ∆cTnT (Table 3). Out of interest, while ∆cTnT was not different between groups, adults played at a higher speed than children and, curiously, speed was indeed positively associated with ∆cTnT (see Table 1). In this regard, although adults are expected to run faster given their anthropometric and structural advantages, this inconsistency suggests a potential interaction between participants’ age and exercise load that should be further investigated in future studies. We also found that the players with higher baseline cTnT concentrations were those who, during the match, performed at a higher relative HR (% HRmax); however, this association was not true for absolute HR (bpm). By contrast, the exercise-induced elevations of cTnT correlated with absolute, but not relative, HR (see Table 3). On the one hand, since HRmax was age-calculated, the baseline differences might be originate from the positive correlation found between basal cTnT and age. On the other hand, although previous research was inconclusive regarding the association between exercise HR and ∆cTnT [3,4,5,28,31], we found higher releases in participants achieving higher absolute heart rates. This finding adds to previous knowledge, supporting the idea that exercise load metrics, such as those recorded in training watches or smartphone apps, might be consulted to identify physiological elevations of cTnT related to exercise.

The strengths of the present study were that it is the first study to report values of cTnT after a football 7 match in both healthy children and adults. To the best of our knowledge, this is also the first study comparing adults with children instead of adolescents. In this regard, our results show the need for future studies assessing whether the cTnT elevation after exercise differs between children and adults. In this study, we included a convenience sample of male football players, and this implied a small sample size, particularly in the adult group. Additionally, other factors such as dietary intake or body composition were not assessed in this study [32]. Although the sample size in this study allowed us to demonstrate the elevation of cTnT after a football 7 match in children and adult players, larger numbers would have permitted us to investigate the interaction and partial correlations between cTnT elevations and other characteristics, including performance and lifestyle variables. Another limitation was that we could not incorporate a third blood sample at 24 h after exercise and, hence, we had no data to confirm whether all participants returned to baseline concentrations during the subsequent day, as has been reported in previous studies [3,4]. For these reasons, future studies should address our limitations by encompassing larger samples, wider ranges of age, and a more exhaustive set of blood measurements. Finally, in this study we only measured cTnT, and other biomarkers such as cTnI or NT-proBNP might be used in future studies to provide a more complete overview of the phenomenon.

## 5. Conclusions

In conclusion, this is the first study proving that a football 7 match is enough stimulus to induce elevations of cTnT, exceeding the URL in ~70% of a healthy cohort of participants. Furthermore, adults had higher basal concentrations, although the magnitude of cTnT elevations was comparable between children and adults. Finally, it seems that that the elevations of cTnT could be associated to exercise load but not to participant characteristics.

## Figures and Tables

**Table 1:** Summary of participants’ characteristics and exercise load.

Variable	Children (*n* = 24)	Adults (*n* = 12)	All (*n* = 36)	Between-Groups
Participant characteristics				
Age (years)	10.7 ± 1.6	37.5 ± 12.7	19.6 ± 14.7	*p* < 0.001
Body height (cm)	146 ± 14.8	177 ± 5.72	157 ± 19.1	*p* < 0.001
Body mass (kg)	41.3 ± 15.4	79.5 ± 7	54.0 ± 22.4	*p* < 0.001
Training experience (years)	4.6 ± 1.7	23.6 ± 14.5	9.4 ± 10.9	*p* = 0.007
Training frequency (days/week)	2.9 ± 1.2	3.2 ± 0.8	3 ± 1.1	*p* = 0.73
Training volume (hours/week)	4.6 ± 2.6	4.9 ± 2.5	4.7 ± 2.5	*p* = 0.55
Exercise load				
Distance (m)	5970 ± 722	5490 ± 540	5810 ± 697	*p* = 0.17
SPpeak (km/h)	23.5 ± 2.2	27.1 ± 1.9	24.7 ± 2.7	*p* < 0.001
SPmean (km/h)	5.6 ± 0.7	6 ± 0.7	5.7 ± 0.7	*p* = 0.04
HRpeak (bpm)	202 ± 6	188 ± 7	197 ± 9	*p* < 0.001
rHRpeak (% HRmax)	100 ± 3	105 ± 6	102 ± 5	*p* = 0.14
HRmean (bpm)	161 ± 19	158 ± 12	160 ± 16	*p* = 0.5
rHRmean (% HRmax)	80 ± 9	88 ± 8	83 ± 10	*p* = 0.084

Values are expressed as mean ± standard deviation. SPpeak, peak speed; SPmean, mean speed; HRpeak, peak heart rate; rHRpeak, peak relative heart rate; HRmean, mean heart rate; rHRmean, mean relative heart rate.

**Table 2:** Values of cardiac troponin T (cTnT) (ng/L).

	Pre	Post	Delta	Within-Subjects
All (*n* = 36)	5.20 (3.65, 12.7)	21.1 (4.67, 200)	13.7 (0.450, 194)	*V* = 666, *p* < 0.001
Children (*n* = 24)	4.64 (3.65, 12.7)	22.9 (4.67, 200)	16.4 (0.450, 194)	*V* = 300, *p* < 0.001
Adults (*n* = 12)	6.54 (4.88, 11.7)	18.6 (8.31, 60.2)	12.4 (0.500, 53.7)	*V* = 78, *p* = 0.001
Between-groups	*W* = 36, *p* < 0.001	*W* = 158, *p* = 0.65	*W* = 171, *p* = 0.37	

Values are expressed as median (range) (ng/L).

**Table 3:** Table of correlations.

Variable	cTnT Pre (ng/L)	∆cTnT (ng/L)
∆cTnT (ng/L)	*r*_s_ = −0.12, *p* = 0.523	
Participant characteristics		
Age (years)	*r*_s_ = 0.67, *p* < 0.001	*r*_s_ = −0.23, *p* = 0.21
Body height (cm)	*r*_s_ = 0.64, *p* < 0.001	*r*_s_ = −0.23, *p* = 0.2
Body mass (kg)	*r*_s_ = 0.55, *p* = 0.001	*r*_s_ = −0.35, *p* = 0.052
Training experience (years)	*r*_s_ = 0.56, *p* = 0.001	*r*_s_ = −0.16, *p* = 0.37
Training frequency (days/week)	*r*_s_ = 0.32, *p* = 0.072	*r*_s_ = −0.09, *p* = 0.64
Training volume (hours/week)	*r*_s_ = 0.2, *p* = 0.27	*r*_s_ = −0.2, *p* = 0.29
Exercise load		
Distance (m)	*r*_s_ = −0.133, *p* = 0.465	*r*_s_ = 0.59, *p* < 0.001
SPpeak (km/h)	*r*_s_ = 0.32, *p* = 0.074	*r*_s_ = 0.17, *p* = 0.37
SPmean (km/h)	*r*_s_ = 0.2, *p* = 0.28	*r*_s_ = 0.59, *p* < 0.001
HRpeak (bpm)	*r*_s_ = −0.18, *p* = 0.33	*r*_s_ = 0.43, *p* = 0.013
rHRpeak (% HRmax)	*r*_s_ = 0.36, *p* = 0.044	*r*_s_ = 0.21, *p* = 0.25
HRmean (bpm)	*r*_s_ = 0.29, *p* = 0.11	*r*_s_ = 0.42, *p* = 0.016
rHRmean (% HRmax)	*r*_s_ = 0.52, *p* = 0.002	*r*_s_ = 0.29, *p* = 0.11

cTnT Pre, cTnT concentration at baseline; ∆cTnT, elevation of cTn from baseline to 3 h post exercise; SPpeak, peak speed; SPmean, mean speed; HRpeak, peak heart rate; rHRpeak, peak relative heart rate; HRmean, mean heart rate; rHRmean, mean relative heart rate.
